# Non-radiologist-performed point-of-care ultrasonography in paediatrics — European Society of Paediatric Radiology position paper

**DOI:** 10.1007/s00247-020-04843-6

**Published:** 2020-11-19

**Authors:** Rick R. van Rijn, Samuel Stafrace, Owen J. Arthurs, Karen Rosendahl, on behalf of the European Society of Paediatric Radiology, Maria Argyropoulou, Pablo Caro-Dominguez, Franz W. Hirsch, Lauerma Kirsi, Ola Kvist, Amaka C. Offiah, Cathy M. Owens, Philippe Petit, Maria Raissaki, Andrea Rossi

**Affiliations:** 1 Department of Radiology and Nuclear Medicine, Emma Children’s Hospital–Amsterdam UMC, University of Amsterdamn, Meibergdreef 9, 1105 AZ Amsterdam Zuid-Oost, the Netherlands; 2 Department of Diagnostic Imaging, Sidra Medicine, Doha, Qatar; 3 Weill Cornell Medicine, Doha, Qatar; 4 Great Ormond Street Hospital for Children, NHS Foundation Trust, London, UK; 5 UCL GOS Institute of Child Health, London, UK; 6 NIHR Great Ormond Street Hospital Biomedical Research Centre, London, UK; 7 Department of Radiology, University Hospital of North Norway, Tromsø, Norway; 8 The Arctic University of Norway, Tromsø, Norway

**Keywords:** Bedside, Children, Non-radiologist, Nonspecialist, Position statement, Ultrasound

## Abstract

Non-radiologist point-of-care ultrasonography (US) is increasingly implemented in paediatric care because it is believed to facilitate a timely diagnosis, such as in ascites or dilated renal pelvicalyceal systems, and can be used to guide interventional procedures. To date, all policy statements have been published by non-radiologic societies. The European Society of Paediatric Radiology hereby issues a position statement on paediatric non-radiologist point-of-care US from the point of view of those leading on children’s imaging, i.e. paediatric radiologists. In this position statement, we will address the boundaries, education, credentialing, quality control, reporting and storage of images in paediatric practice.

## Introduction

Historically, ultrasonography (US) has been part of an integrated imaging strategy as performed by radiologists, but the method has also been used by gynaecologists, cardiologists and ophthalmologists, amongst others, as part of their clinical care. However, with the advent of smaller, cheaper, portable systems, other specialties have taken an interest in the use of US. This development was foreseen by the European Society of Radiology (ESR) in its 2009 position paper on US, stating: “Turf battles about the use of US continue to grow as more and more specialists are claiming US as part of their everyday’s work, and the position of radiologists is progressively further undermined” [1]. In the past years, all major medical imaging companies have added handheld devices to their spectrum of imaging modalities [2]. The use of US by non-radiologists has become known as point-of-care ultrasonography (POCUS). As discussed previously in this journal, point-of-care US actually is a misnomer as radiologists also provide point-of-care US services [3]. We will therefore use the terminology non-radiologist point-of-care US throughout this position statement. The last decade has seen a significant uptake of non-radiologist point-of-care US, although to date mostly in adult and emergency medicine. As early as 1990, the American College of Emergency Physicians issued a statement on non-radiologist point-of-care US in emergency medicine [4]. Although it can be argued that it is a relatively new development, it already has been suggested that non-radiologist point-of-care US should be seen as the fifth pillar next to the four historical pillars of inspection, palpation, percussion and auscultation [5].

It goes without saying that there are several potential advantages to non-radiologist point-of-care US. First of all, it brings imaging to the bedside, directly at the patient-attending physician level. This means that imaging can be expedited. It also helps improve urgent diagnosis by avoiding the loss of clinical information via the normal referral pathway. Second, the implementation of non-radiologist point-of-care US can also overcome the problem of staff shortages. This applies equally to countries with a shortage of radiologists or institutions unable to provide a 24/7 bedside service because of radiologist or US technician staffing. Lastly, implementing non-radiologist point-of-care US can lead to cost efficiency and consequently to a decrease in overall health care costs [6]. On the other hand, it can be argued, and personal experiences have shown, that it can lead to unnecessary additional studies due to equivocal non-radiologist point-of-care US results, to repeat examinations with decreased patient compliance (especially in young children) and anxious parents, and to missed diagnoses due to the overconfidence or limited experience of the non-radiologist point-of-care US provider [7].

These advantages have led to an increased uptake by non-imaging specialties where non-radiologist point-of-care US has even become part of specialist training and undergraduate education curricula [8–12]. In the last few years, several societies, e.g., the American Academy of Pediatrics (AAP), the American College of Emergency Physicians, the Society of Hospital Medicine, and the World Federation for Ultrasound in Medicine and Biology, have issued position statements on non-radiologist point-of-care US [13–16]. Although the European Society of Radiology published a position statement on portable US devices, to our knowledge, there are no position statements published by paediatric radiologic societies [2]. We, as members the European Society of Paediatric Radiology (ESPR), believe the time has come to issue a position statement on paediatric non-radiologist point-of-care US from the paediatric radiologist’s point of view.

## Minimum requirements for non-radiologist point-of-care US

The process of implementing non-radiologist point-of-care US consists of two different domains:

1. Education, credentialing and quality control.
2. Practical implementation in daily routine consisting of performing, reporting and storing non-radiologist point-of-care US examinations.

### Education

With the increased uptake of non-radiologist point-of-care US comes an increased need for education. For some specialties this has already become mandatory, e.g., in 2001 the Accreditation Council for Graduate Medical Education (ACGME) mandated that all emergency medicine residents should be trained in non-radiologist point-of-care US. Despite this mandatory training, studies have shown that there is a difference in training opportunities between facilities [17]. In the United States, the Society for Clinical Ultrasound Fellowships (https://eusfellowships.com/) lists 15 institutions that offer a fellowship in paediatric non-radiologist point-of-care US. Besides these official fellowships, there are commercial initiatives, including by manufacturers, that offer hands-on or online tutorials. It is of interest to note that many of these courses are organised and given without recognisable involvement from radiologists. The focus of education should not only be on applying non-radiologist point-of-care US as a technique, i.e. the acquisition of adequate images, as this inherently increases the risk of reducing it to a gimmick. There should also be a strong focus on the indications and contraindications of non-radiologist point-of-care US, on understanding the limitations of this method depending on the indication, as well as on incorporating findings in subsequent imaging examinations and in the patients’ treatment plans.

As education is an essential part of the success of implementing paediatric non-radiologist point-of-care US, and collaboration between first-line caregivers and paediatric radiologists is key to optimise patient care, we believe that paediatric radiologists should become involved in non-radiologist point-of-care US education. Such involvement of paediatric radiologists raises the question of the critical mass of these specialists needed to complete this education plan. We believe that an increase in the number of paediatric radiologists is required to be able to provide these services.

Ideally, this education should be offered in a modern blended learning environment where, with the use of a flipped classroom, hands-on training and online courses (e.g., a massive open online course), an optimal educational environment is created for the trainee. If clinicians are properly trained in non-radiologist point-of-care US they will not only be able to provide proper patient care, but they will also be aware of the limits of their capabilities and know when to consult a radiologist.

### Credentialing

In the European Union, most, if not all, countries adhere to the European Training Curriculum for Radiology, which describes requirements to be met on three levels of expertise: undergraduate radiology, radiology and subspecialisation in radiology [18]. Based on the criteria in these documents, trainees can be credentialed at the various levels of expertise. This expertise can be certified by sitting a general European radiology exam leading to the European Diploma in Radiology (EDiR) [19]. A next step would be to obtain a subspecialty diploma, for example in paediatric radiology (EDiPR) [20] or paediatric neuroradiology (EDiPNR). It is clear that a non-radiologist will not be able to meet the demands set in these diplomas nor should we expect them to. However, there is a need for credentialing non-radiologists who want to become involved in non-radiologist point-of-care US. Whereas the AAP in its policy statement states that credentialing is important, it doesn’t specify how this should be done or what levels of expertise should be met to achieve it. The ACGME and the American Board of Emergency Medicine have defined competency levels for emergency medical training, one of which describes the sequential learning process for non-radiologist point-of-care US [21]. This could serve as a foundation for future credentialing, where the level of expertise to be achieved should be realistically set keeping in mind the overall aim of non-radiologist point-of-care US. Although ideally the aim should be a pan-European credentialing system, for now this seems to be a bridge too far to cross. Therefore, credentialing should be organised on a national or even an institutional level. With respect to a credentialing board it seems logical that, as paediatric radiologists are the experts in the field, they should be actively involved.

### Quality control

Radiologists are mandated to follow dedicated post-academic education to maintain proficiency in radiologic procedures in their subspecialty. As non-radiologists are not bound by the guidelines dictating radiology training, facilities that allow non-radiologist point-of-care US should have some form of quality control in place. Multiple societies already have published non-radiologist point-of-care US guidelines that include paragraphs on quality control. Part of the quality control program should focus on quality and updating of equipment, diagnostic quality of technique/applied protocol (e.g., the number and specifications of views per indication), diagnostic quality of the acquired images (e.g., appropriateness of settings), appropriateness of interpretation of imaging findings and feedback on the clinical impact of the study. Ideally, institutions or societies should have a committee of experts to carry out quality control. We believe there should be a place for a paediatric radiologist in such a committee as they are the true experts in the use of paediatric US.

### Non-radiologist point-of-care US indications

In line with a remark in the AAP position statement, the ESPR believes non-radiologist point-of-care US should be limited to guiding specific interventions, such as line placement and suprapubic punctures, or to those studies that are performed to promptly answer specific diagnostic yes/no questions [22]. This approach contrasts the comprehensive US examination performed by paediatric radiologists as an integrated part of an imaging work-up, including a detailed assessment of the actual region, with colour and spectral Doppler, elastography or intravenous contrast added as appropriate. Within Europe, the training should be in line with the European Training Curriculum for Radiology where, preferably for paediatric radiology, subspecialty training has been undertaken [23, 24]. The question arises of which studies can be performed as non-radiologist point-of-care US after a limited period of training and supervision. All radiologists will have several anecdotal cases in which non-radiologist point-of-care US missed the diagnosis thus leading to a delay in the diagnosis and potential damage to the patient. However, this remains anecdotal as there are no studies into the true extent, if at all present, of this problem. Non-radiologist point-of-care US can be divided into five domains, as specified by the American College of Emergency Physicians, covering a wide range of indications (Table 1). Although this division into domains is useful, it does not prioritize which aspects should be taught to and performed by a non-radiologist. Using the Delphi Technique, an international team of members of the Paediatric Emergency Medicine POCUS Network (P2Network) determined which indications should be incorporated in its fellowship training (Table 2) [22]. An almost overlapping list of indications can be created by combining the publications from the Society of Hospital Medicine and the Society for Academic Emergency Medicine (Table 3) [15, 23]. Both these lists, however, go beyond a simple diagnostic yes/no question and require in-depth knowledge of clinical radiology and diagnostic and interventional US. An example is diagnosing appendicitis, which may indeed be straightforward to diagnose if the appendix is easy to visualise but, in daily practice, this is often difficult and can be challenging even for advanced radiology residents. A similar case can be made for the diagnosis of intussusception. In very straightforward cases, which meet all the clinical criteria for the diagnosis, it is a relatively simple diagnosis with well-known false-positive and false-negative findings. However, only about 30% of paediatric cases present with the classic clinical criteria, and the imaging findings are also used to decide the method of treatment; therefore, the use of non-radiologist point-of-care US could even lead to a delay in treatment [24]. In several domains, including acute abdominal pain in children, age, symptoms and initial US findings will guide the paediatric radiologist to modify the technique, potentially assess other areas like the inguinal canal or the lung bases and pay attention to detail to reach a correct diagnosis. Therefore, in a classic clinical scenario, non-radiologist point-of-care US could also lead to additional costs and potentially delay the diagnostic/treatment process. With respect to interventional procedures, there has been a strong shift toward subspecialisation in this field, exemplified by abscess drainage, where, unless very superficial, the procedure not only requires a dedicated skill set but also a dedicated environment and interventional equipment. Both will not be readily available to paediatricians performing non-radiologist point-of-care US.

**Table 1:** American College of Emergency Physicians classification of non-radiologist point-of-care ultrasound (US) [14]

Category	Description
Resuscitative	US used as directly related to an acute resuscitation
Diagnostic	US used in an emergent diagnostic imaging capacity
Symptom- or sign-based	US used in a clinical pathway based upon the patient’s symptom or sign (e.g., shortness of breath)
Procedure guidance	US used as an aid to guide a procedure
Therapeutic and monitoring	US used in therapeutics or in physiological monitoring

**Table 2:** Non-radiologist point-of-care US applications to include in training as presented by the Paediatric Emergency Medicine by Delphi Technique POCUS Network (P2Network) [22]

Application	Percentage of experts who ranked each application as “extremely/very important”
*Round 1*	
Identify free peritoneal fluid in trauma	100
Identify non-traumatic pericardial effusion	100
Identify pericardial effusion in trauma	100
Identify haemothorax	98
Identify pleural fluid/effusion	98
Identify pneumothorax	98
Identify cardiac standstill	96
Abscess incision and drainage	94
Identify abscess	94
Central line placement	91
Evaluate cardiac function	88
Identify cellulitis	88
Identify intussusception	87
Identify intrauterine pregnancy	85
Identify soft-tissue foreign body	85
Assess bladder volume	83
Identify lung consolidation	83
Peripheral intravenous access	81
*Round 2*	
Foreign body localizations and removal	89
Identify pulmonary oedema	84
Pericardiocentesis	84

**Table 3:** Indications for non-radiologist point-of-care US and for paediatric radiologic studies based on the combined position statements of the Society of Hospital Medicine (SHM) and the Society for Academic Emergency Medicine (SAEP) [15, 23]

Organ system	Indication for non-radiologist point-of-care US	Indications for paediatric radiologist study
Pulmonary	Pleural effusion Pneumothorax	Alveolar syndromes Interstitial syndromes
Abdominal	Early pregnancy Free fluid Gallbladder—Cholelithiasis Bladder volume Organ size (liver, kidney, spleen)	Appendicitis Gallbladder – Cholecystitis Dilated pelvicalyceal systems (hydronephrosis) Intussusception^a^ Pyloric stenosis
Vascular		DVT AAA^b^
Musculoskeletal	Abscess Cellulitis Joint effusions	Foreign bodies Fracture
Intervention	Arterial line placement Arthrocentesis CVC placement Lumbar puncture Paracentesis	Abscess drainage^c^
Multisystem^d^		
*Hypotension and shock*	Cardiac^e^ Pulmonary Abdominal free fluid	DVT
*Resuscitation*	Cardiac Pulmonary	
*Dyspnoea*	Cardiac Pulmonary	DVT
*Acute renal failure*	Renal Bladder Central venous pressure Pulmonary	

^a^The use of non-radiologist point-of-care US in diagnosing intussusception will add to the total number of examinations leading to a delay in the diagnostic and treatment process

^b^In paediatrics, this is an extremely rare diagnosis that needs a specialist’s imaging and evaluation

^c^Except for very superficial abscesses it requires specific knowledge, a dedicated environment and interventional materials

^d^Only in the acute setting, otherwise these multisystem studies should preferably be performed by a (paediatric) radiologist

^e^Cardiac non-radiologist point-of-care US, according to the position statements of the SHM and the SAEP, consists of the assessment of atrial size, central venous pressure, chamber hypertrophy, gross valvular abnormalities, left ventricle assessment, pericardial effusion and right ventricle assessment. As this is outside the scope of normal paediatric radiological casework, the ESPR has chosen not to take a position with respect to these examinations

*AAA* abdominal aortic aneurysm, *CVC* central venous catheter, *DVT* deep venous thrombosis

## Storage

With the advent of digital electronic health record (EHR) systems, a paradigm shift in image storage is imminent. Radiology has for a long time been in the lead with the installation of dedicated picture archive and communication systems (PACS). Other clinicians are currently looking for image storage as well. Unfortunately, conventional PACS are not designed to cater to the needs of other specialists or to deal with images that are not DICOM (Digital Imaging and Communications in Medicine). The result is that there is a risk that images from non-radiologist point-of-care US are not consistently stored and displayed in the EHR. This leads to an unacceptable fragmentation of medical imaging where the chronology is lacking and clinicians and radiologists don’t have access to a complete overview of patient records.

Additionally, the current radiology information systems and PACS are specifically designed to serve the workflow of the departments of radiology and nuclear medicine. By design they support a large number of different roles, e.g., planning and booking and radiology workflows within the department of radiology. However, this also implies many of these systems cannot be used to support non-radiologist point-of-care US practice. In contrast, newer developments have led to enterprise imaging platforms, i.e. an open PACS with a vendor neutral archive capable of combining demographic patient information with a multitude of imaging devices and modalities from within the institute or even outside its network and including import from outside imaging. This whole process can be done in the background and can be based on subspecialty practice-based workflows. Once this is in place, access to an EHR where all medical imaging is presented in a chronological and organised order will lead to an increased level of patient care. Such a development will also be needed for reporting (see below) and billing non-radiologist point-of-care US in a timely and orderly fashion.

### Reporting

Whereas in comprehensive US it is obvious that a timely and accessible report is key for good patient management, this is not the case for non-radiologist point-of-care US. For example, the AAP policy statement says, “Details of the point-of-care ultrasonography examination must be documented at the time of performance in the medical record,” and the Society of Hospital Medicine policy statement says, “Documentation can occur through a standalone note or as part of another note, such as a progress note” [15, 25]. From a radiologist’s perspective, these recommendations are insufficient as they don’t specifically describe that the report should be easily findable and in the correct chronology by other health care providers. Incorporating the findings in a progress note will almost definitely lead to a loss of information [26, 27]. Progress notes or scanned documents are unstructured data from which it is difficult, if not impossible, to retrieve relevant information. Moreover, non-radiologist point-of-care US performers, similar to radiologists, may incorporate structured reporting in their clinical practice and should consider its different forms, its specific targets and its specific demands [28]. Consequently, it should be mandatory for clinicians to report the non-radiologist point-of-care US studies much the same as radiologists treat their reports, structured in a separate findable report and not hidden in clinical notes or a letter.

## Conclusion

It is clear that non-radiologist point-of-care US provides many apparent solutions to resource-limited institutions. The ESPR standpoint is that we support non-radiologist point-of-care US where good training and an accreditation and governance structure exists. This will ensure adequate clinical practice, keeping in mind that more paediatric radiologists are needed to ensure the quality of the programme and the quantity of teachers. Additionally, the ESPR should work further to establish minimum reference standards.

The ESPR believes that non-radiologist point-of-care US should not be implemented without a governance infrastructure, as it could lead to erroneous diagnoses, poor patient outcomes and health care practitioner litigation. It is up to the individual operator to ensure that they are trained to international standards. The ESPR is committed to providing high-level, structured teaching courses to such avail (education, training and credentialing). To quote the European editor of this journal on the topic of non-radiologist point-of-care US, “Regardless of who does it and where it is done, let’s do it well!” [3].
